# Smartphone apps for orthopaedic sports medicine – a smart move?

**DOI:** 10.1186/s13102-015-0017-6

**Published:** 2015-10-12

**Authors:** Seng Juong Wong, Greg A. Robertson, Katie L. Connor, Richard R. Brady, Alexander M. Wood

**Affiliations:** 1 University of Edinburgh, College of Medicine and Veterinary Medicine, 7/3 West Nicolson Street, Edinburgh, EH8 9DA UK; 2 Department of Trauma and Orthopaedics, Royal Infirmary Edinburgh, Edinburgh, UK; 3 Department of General Surgery, Kirkaldy, Fife UK; 4 Department of Colorectal Surgery, Western General Hospital, Edinburgh, UK; 5 Department of Orthopaedics, Wansbeck Hospital, Ashington, UK

**Keywords:** Orthopaedic sports medicine, Smartphone, Apps

## Abstract

**Background:**

With the advent of smartphones together with their downloadable applications (apps), there is increasing opportunities for doctors, including orthopaedic sports surgeons, to integrate such technology into clinical practice. However, the clinical reliability of these medical apps remains questionable. We reviewed available apps themed specifically towards Orthopaedic Sports Medicine and related conditions and assessed the level of medical professional involvement in their design and content, along with a review of these apps.

**Method:**

The most popular smartphone app stores (Android, Apple, Blackberry, Windows, Samsung, Nokia) were searched for Orthopaedic Sports medicine themed apps, using the search terms; Orthopaedic Sports Medicine, Orthopaedics, Sports medicine, Knee Injury, Shoulder Injury, Anterior Cruciate Ligament Tear, Medial Collateral Ligament Tear, Rotator Cuff Tear, Meniscal Tear, Tennis Elbow. All English language apps related to orthopaedic sports medicine were included.

**Results:**

A total of 76 individual Orthopaedic Sports Medicine themed apps were identified. According to app store classifications, there were 45 (59 %) medical themed apps, 28 (37 %) health and fitness themed apps, 1 (1 %) business app, 1 (1 %) reference app and 1 (1 %) sports app. Forty-nine (64 %) apps were available for download free of charge. For those that charged access, the prices ranged from £0.69 to £69.99. Only 51 % of sports medicine apps had customer satisfaction ratings and 39 % had named medical professional involvement in their development or content.

**Conclusions:**

We found the majority of Orthopaedic Sports Medicine apps had no named medical professional involvement, raising concerns over their content and evidence-base. We recommend increased regulation of such apps to improve the accountability of app content.

## Background

The market of health-related smartphone downloadable applications (apps) is rapidly expanding, with approximately 1,000 new apps released each month [1] and 142 million annual downloads predicted by 2016 [2]. At present, there are more than 100,000 healthcare related apps [3] and this sector is predicted to rise by 25 % per annum over the next five years [4].

Smartphone usage is popular amongst health care professionals, with one study reporting 84 % orthopaedic care providers in the USA owned a Smartphone and 53 % using it in clinical practice [5]. The range of smartphone applications available has been reported in various specialties, including orthopaedics [5, 6], neurosurgery [7, 8], plastic surgery [9], general surgery [10], colorectal surgery [11], bariatric surgery [12], hernia surgery [13], radiology [1], pain medicine [14], dermatology [15], infectious diseases [16] and microbiology [17].

Within the United States, over 3,000 clinicians have been registered as Orthopaedic Sports Medicine practitioners and six of the commonest orthopaedic procedures fall within the category of Orthopaedic Sports Medicine [18]. As such, there is a significant demand for information relating to Orthopaedic Sports Medicine for both clinicians and patients alike. Notably, previous studies have found internet information relating to Orthopaedic Sports Medicine to be limited and of variable quality [19]. Smartphone apps have been advocated as the contemporary modality to convey such information [20, 21]. Previous studies have assessed the validity of Smartphone apps relating to specific aspects of sports medicine such as injury prevention [22]. However, there is currently no comprehensive study investigating the current provision of apps in the field of Orthopaedic Sports Medicine.

There are pertinent concerns regarding the lack of medical professional involvement in app design [11–13, 15, 17], and the reliability and accuracy of app content for healthcare related apps in a number of specialities [1, 11–15, 17]. Whilst regulations imposed by the US Food and Drug Administration (FDA) exist for medical smartphone apps which directly influence patient treatment [23], most medical smartphone apps are not formally evaluated under the current guidance [24].

This study aimed to identify contemporary smartphone apps relating to Orthopaedic Sports Medicine, assess the level of medical professional involvement in their design, and provide an overview of the related apps available.

## Method

Six major online mobile platform application stores (Android, Apple, Blackberry, Nokia, Samsung, Windows) were searched for apps relating to Orthopaedic Sports Medicine by a single author on 23^rd^ July 2015. The search terms were based on the commonest Orthopaedic Sport Medicine conditions encountered by sports surgeons [19] and included; Orthopaedic Sports Medicine, Orthopaedics, Sports medicine, Knee Injury, Shoulder Injury, Anterior Cruciate Ligament Tear, Medial Collateral Ligament Tear, Rotator Cuff Tear, Meniscal Tear, Tennis Elbow.

Data was generated from the overview pages of the apps provided by the developer. All links advertised on the overview page were also accessed and reviewed to fully ascertain the degree of professional involvement in the app design. Data recorded for every app included app stores’ category of the application, description of the app, implied target audience, documentation of medical professional or organisation involvement, evidence referenced, average rating, number of ratings, publisher information, date of last updates, commercial content and cost [prices converted to British pounds sterling]. All app links to publisher pages were followed to establish authorship, referenced evidence and links to commercial products. Commercial intent was judged by whether the app or the publisher page: had links to private clinics, software developers, or medical technology companies. Only one commercial interest was counted per app. Apps identified were then classified into categories (Education, Exercise, Journal, Conference, etc) and summarized for clarity.

Apps relating to sports medicine but with no relation to orthopaedic specialty were excluded. Similarly non-English language applications, games and wallpaper applications were excluded. When repeat applications were found, these were marked as duplicates and only one version of the app was counted.

Uni-Variate Statistical Comparisons between categorical variables were performed using Chi Squared Test on Statistical Package for Social Science (SPSS). The significance level was set at *p* < 0.05.

## Results

**Fig. 1:**
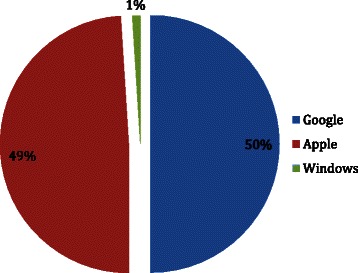
Apps Distribution by App Stores

This search generated a total of 1089 ‘hits’, of which 994 apps were excluded due to unrelated content (games, wallpapers, non-English language apps), and 19 apps were discounted due to repetition. Hence, a total of 76 apps were included and analyzed. Thirty eight (50 %) apps were identified on Google’s Android store, 37 (49 %) apps were identified on Apple’s App store, one (1 %) app on Windows platform and none on the other app stores. (see Fig. 1).

**Fig. 2:**
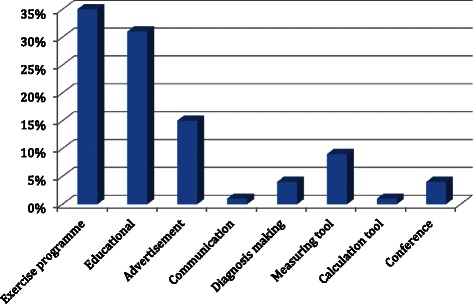
Categories of Smartphone Applications

According to the App stores classifications, there were 45 (59 %) medical themed apps, 28 (37 %) health and fitness themed apps, one (1 %) business app, one (1 %) reference app and one (1 %) sports app. Apps were categorised into different categories (see Fig. 2). The overall apps search results are summarized in Table 1 and a list of ten most popular apps downloaded are shown in Table 2.

**Table 1:** Categorical overview of the sports medicine apps identified with respect to their cost, medical professional involvement and consumer ratings

Category	Target audience	No. of Apps	Price range [Mean]	Medical professional involvement	No. of Apps with ratings	Customer rating (range)
OVERALL	Patients/HCW	76	£0–£69.99 [£6.77]	30/76 (39 %)	39/76 (51 %)	Mean Score: 3.76 (1–5) Total reviewers: 471
Exercise programme	Patients	27	£0–£6.1 [£2.37]	10/27 (37 %)	14/27 (52 %)	Mean Score: 3.86 (1–5) No. of reviewers:202
Education *Subcategories*	Patients/HCW	23	£0–£2.33 [£1.72]	9/23 (39 %)	15/23 (65 %)	Mean Score: 3.8 (2–5) No. of reviewers: 211
*HCW education*	HCW	13	£0–£2.33 [£2.00]	4/13 (31 %)	5/13 (38 %)	Mean Score: 3.86 (3–5) No. of reviewers: 122
*Patient education*	Patients	10	£0–£0.85 [£0.85]	5/10 (50 %)	10/10 (100 %)	Mean Score: 3.77 (2–5) No. of reviewers: 89
Advertising *Subcategories*	Patients/ HCW	11	£0 [£0]	6/11 (55 %)	6/11 (55 %)	Mean Score: 4.3 (3–5) No. of reviewers: 34
*Patient advertising*	Patients	9	£0 [£0]	6/9 (67 %)	5/9 (56 %)	Mean Score: 4.55 (4–5) No. of reviewers: 29
*HCW advertising*	HCW	2	£0 [£0]	Nil (0 %)	1/2 (50 %)	Mean Score: 2.5 (1–5) No. of reviewers: 5
Communication tool	HCW	1	£0 [£0]	1/1 (100 %)	Nil (0 %)	- No. of reviewers: 0
Measuring tool	HCW	7	£0.61–£7.5 [£3.67]	2/7 (29 %)	4/7 (57 %)	Mean Score: 2.4 (1–5) No. of reviewers: 34
Calculation tool	HCW	1	£2.92	Nil (0 %)	Nil (0 %)	- No. of reviewers: 0
Conference	HCW	3	£0 [£0]	Nil (0 %)	Nil (0 %)	- No. of reviewers: 0
Diagnosis making	Patients/HCW	3	£0–£69.99 [£39.99]	2/3 (66 %)	Nil (0 %)	- No. of reviewers: 0

No. = Number. HCW: Health Care Worker, N/A: Not Applicable

**Table 2:** Top ten most downloaded apps and what they do

Name of app	Description of the app
Optech live	Provides Orthopaedic Specialists with easy access to the latest Stryker Orthopaedics surgical techniques
Meniscal Tear	Contains animated rehabilitation exercises for patients following meniscal tears
Anterior Cruciate Ligament	Contains strengthening exercises, plyometrics, balancing exercises for patients with ACL injuries
Tennis Elbow	Contains causes and symptoms of tennis elbow along with animated rehabilitation exercises
Patellar Tendonitis	Contains causes, symptoms and treatment for patellar tendinitis along with animated rehabilitation exercises
Sports Injury Clinic	Provides information on over 100 sports injuries covering symptoms, an explanation of the injury and treatment methods.
Orthopaedics Today Europe	Provides access to the latest issue of Orthopaedics Today Europe
MediGrip	Provide healthcare workers with the most relevant and interesting results in Orthopaedic Sports Medicine research.
Knee Goniometer	An accelerometer based knee goniometer for use in patients with Orthopaedic Sports Medicine Injuries
Throw Like a Pro	Provides an overview of baseball throwing injuries, with and medical advice for injury prevention

Forty-nine (64 %) of the apps were available for download free of charge. For those that charged access, the prices ranged from £0.69 for a rehabilitative exercise programme app to £69.99 for a diagnosis making and management app. The mean cost of chargeable apps was £6.77.

Seven (9 %) apps were updated in 2015 (the latest being 9^th^ February), 16 (21 %) apps were updated in 2014, 20 (26 %) apps in 2013, 14 (18 %) apps in 2012, 13 (17 %) apps in 2011, five (7 %) in 2010 (the oldest update being 24^th^ July 2010) and one (1 %) app did not specify time of up-date.

Thirty-nine (51 %) apps had customer satisfaction ratings. A total of 471 consumers rated these apps. The mean customer satisfaction rating for all the rated apps was 3.76 out of 5. 30 (77 %) of the apps with customer satisfaction ratings were free while 19 (51 %) without ratings were free (*p* < 0.02).

Thirty (39 %) of apps had clearly documented medical professional involvement evident from the overview pages or associated links provided. Twenty (67 %) of the apps with named medical professional involvement were free compared with 29 (63 %) without named medical professional involvement (*p* = 0.74).

Commercial links were present in 29 (38 %) of all apps. Commercial interests included: links to software developers (*n* = 18; 62 %), links to medical technology companies (*n* = 4; 14 %), links to private sports clinics (*n* = 3; 10 %),

### Educational apps for patients and healthcare workers

23 (30 %) of apps focused on providing information relating to Orthopaedic Sports Medicine diagnoses. This category was subdivided into education for patients and education for health-care workers. This group of apps had the highest number of reviewers (*n* = 211).

Thirteen (57 %) apps were designed for the education of Health-care workers. Of these, three (23 %) provided up-to-date information in the sports medicine field. Five (38 %) apps were knowledge-based providing information ranging from anatomy to surgical knowledge. One (8 %) app provided the technical expertise relating to the use of extracorporeal shockwave therapy. Additionally there were four (31 %) journal apps: the American Journal of Sports Medicine, Arthroscopy, Clinical Journal of Sports Medicine and Orthopaedic Journal of Sports Medicine. These apps allow users to browse abstracts from the relevant journal and download the full article should they subscribe. Three of these (23 %) apps were chargeable with a mean price of £2.00. Only four (31 %) apps had documented medical involvement. Commercial links were present in 4 (31 %) apps. Five (38 %) apps had consumer ratings, with a mean score of 3.86 out of 5.

Ten (43 %) educational apps were for patients. Of these 10 apps, five (50 %) apps provided patients with general information, two (20 %) apps provide customised information leaflets delivered through a secure server, two (20 %) apps provided information on self-diagnosis and management of common sports complaints and one (10 %) app provided illustrations of sports injuries. Only five (50 %) of these apps had named medical professional involvement. Nine (90 %) of the apps were available free. Commercial links were present in 5 (50 %) apps. This category had a total of 89 reviewers, with a mean review of 3.77 out of 5.

### Exercise programme apps for patients

There were 27 (36 %) Exercise programme apps and their target audience was primarily patients. Most of the apps taught users how to perform sets of exercises, ranging from rehabilitative types mainly for post-operative patients, to stretching and strengthening exercises such as plyometrics to prevent common sports injuries. Eleven (41 %) of these apps provided a step-by-step video-assisted guidance on how to perform sets of exercises. Nineteen (70 %) of the apps provided information on the causes, symptoms and mechanism of particular sports medicine diagnosis. Fourteen (52 %) of the apps discussed treatment options. Only 10 (37 %) of the exercise programme apps had named medical involvement, despite the fact that this group had over 200 reviewers with an average rating of 3.8.

Sixteen (59 %) of the exercise programme apps were chargeable, prices ranging from £0.69 to £6.10, with a mean cost of £2.37. Commercial links were present in 14 (52 %) apps. Only fourteen (52 %) apps in this category were rated, with a mean of 14 reviewers per exercise programme app.

#### Advertising apps

Eleven (14 %) apps specifically advertised an Orthopaedic Sports Medicine related product or Orthopaedic Sports Medicine clinic. These apps had little to no information on common sports injuries. These were further subdivided into patient advertisement and healthcare-workers advertisement apps.

Of the 11 apps, nine (82 %) were advertising apps targeted at patients. All nine (100 %) advertised private Orthopaedic Sports Medicine clinics and enable patients to book an appointment with an Orthopaedic Sports Medicine specialist. These advertising apps were all non-chargeable. Of note, six (67 %) of the apps had medical professional involvement. Five (56 %) out of the nine apps had consumer ratings, with a mean score of 4.55 out of 5, from a total of 29 reviewers.

There were two (18 %) advertising apps targeted at healthcare workers. Both apps allow users to view a range of medical products. No stated medical involvement was noted in these two apps and both were non-chargeable.

#### Measuring tool apps

There were seven measuring tool apps identified. Six (86 %) were goniometers for healthcare workers to measure joint range of motion in rehabilitation programmes. One app, Smartjoint, acted as a KT 1000 arthrometer which quantifies Lachman’s test. Two (29 %) apps had commercial links. Medical professional involvement was noted in 29 %. Four (57 %) apps received consumer reviews with a mean score of 2.4 out of 5. Only one of these apps had been validated by a published study [25].

#### Calculation tool app

One (2 %) app, The Perfect ACL, provided a mathematical formula to allow surgeons to calculate the ‘perfect’ Anterior Cruciate Ligament(ACL) graft length in ACL reconstruction. The app claims to be evidence-based. It is chargeable at £2.92 and has no user ratings.

#### Diagnostic tool app

Three (4 %) apps provided step-by-step algorithm to diagnose common orthopaedic sports injuries. One (33 %) app, ‘Shoulder Injuries’, targeted at patients was designed by US-trained, board-certified physicians. Two (67 %) of the diagnosis-making apps were for doctors and they help doctors reach a sports medicine diagnosis algorithmically. One, the 5-min-Sports-Medicine-Consult, aimed to help clinicians reach a diagnosis in five minutes. While the other, @Hand:Sports Medicine focused on the management of orthopaedic sports-related injuries. These apps had the highest cost of all the Orthopaedic Sports Medicine apps at mean of £39.99 respectively. None of these apps had user ratings and only two stated medical involvement.

#### Conference app

Three (4 %) apps provide healthcare workers with information relating to upcoming conferences such as their location and the programme timetable. It also allowed users to identify key lectures and plan schedules for these events. They were all non-chargeable and did not have any medical professional involved in their design.

## Discussion

Smartphone apps are increasingly used in medical practice [26]. This technology has given clinicians the capability to merge information and communication resources into one multipurpose device [16]. The rapid expansion of smartphone usage in the clinical environment is reflected in the rapid growth within the Smartphone healthcare app sector [26]. The content and validity of smartphone applications has been studied in various medical fields [1, 5–9, 11–17]. Here, we describe the current provision of apps within Orthopaedic Sports Medicine. A number of novel and clinically relevant apps were identified; however this study raises a number of concerns relating to the quality of app content and accountability of their developers.

Overall, there are a wide range of apps available targeted to both medical and lay audiences. For patients, the exercise programmes apps available may be clinically relevant. These apps can be used as an adjuvant to patients’ post-operative care where rehabilitative exercises are commonly difficult to explain at the clinic or when visiting a physiotherapist is difficult. These would work towards reducing the burden on already taxed healthcare systems. Of note, previous studies have found that apps can be used effectively to promote exercise programme that results in positive health effects [27], notably decreasing weight [28–30], blood pressure [30] and cholesterol [30]. However, exercise apps related to the field of sports medicine, notably injury prevention, have been found to have limited evidence base with few studies validating their design and purpose [22]. As such, while these exercise apps show much promise, their benefit still needs to be authenticated by well-conducted scientific research.

Another concern is the unregulated production of medical apps as non-medical programmers could easily develop and publish medical-related apps. While this has some benefits of creativity in design of apps, there remains a larger concern regarding the validity of medical information within such apps, as well as the accountability of apps which influence patients’ decision [1, 11–14, 17]..

While named medical professional involvement in Orthopaedic Sports Medicine apps overall was recorded at 39 %, higher than those reported in other specialties [1, 11], only 15 (20 %) apps claimed reference to evidence-based sources of information. This is despite the fact that these apps could influence decisions on patient management. Moreover, these educational apps are not being regulated by the FDA [23].

Patient confidentiality is a concern with the Exercise Programme apps within this study, with patients required to enter health related variables into these apps, to allow them to function appropriately. This essentially involves the uploading of patient confidential information into these applications; however there is no demonstration of strict governance protecting this information. Of note, there are two apps from the Education category in this study which allowed doctors to prescribe customised information sheets containing patient’s x-rays and surgical videos to patients themselves directly through a supposed secure server. These apps had validated ‘Health Insurance Portability and Accountability Act’ (HIPAA) stamps, thus adding security to the transference of such information. We would encourage that apps involving confidential patient information require validation in such form.

At present, the FDA only regulates a subset of smartphone medical applications which can influence clinical diagnosis or practice (e.g. calculation of drug doses). In the current study, the Measuring and Calculation tool apps potentially fall within this category: however they are not regulated by FDA and only one had evidence-based validation [25]. Other apps that have the potential to influence patient management such as the diagnostic tool apps remain unregulated and quality assurance is further weakened by a paucity of stated medical professional involvement. As apps can influence patient management and subsequent outcomes, stricter regulation should be considered for their development in the future.

Further concerns include difficulties for potential users to assess the accuracy of application content prior to purchase. Currently, application stores provide a short description of the app, along with customer ratings. While customer ratings can provide a peer-reviewed source of feedback, 49 % of apps in this study had no rating. Furthermore, 59 % of those rated were based on less than ten users, reducing the reliability of the customer feedback. This remains a common problem amongst medical apps [14, 15]. Medical apps should be regulated to provide clear information regarding nature of app content and relevant qualifications of app developers. This allows potential users to make an informed assessment of an application’s reliability prior to purchase.

Another potential solution is through peer-review of such applications via independent online publications, such as MedicalAppJournal [31], iMedicalApps [32] and Toporthoapps.com [33]. In the UK, patients can use the NHS Choices Health Apps Library [34] for a list of NHS-endorsed medical apps to manage their health. Similarly, the US National Library of Medicine Apps [35] provide US patients with a list of endorsed medical apps. This allows patients to purchase from a list of relatively safe and quality-assured apps.

Another method may be through the creation of a recognised authentication stamp from a formal organisation to be issued to apps that meet certain standards. The ‘Health on the Net’ Foundation, is a non-profit, non-governmental organization, which promotes the deployment of useful and reliable internet health information for patients and health professionals [36]. It has established a ‘Health On the Net’ Code to facilitate this [36]. This has been previously been assessed by peer review and has been found to act as a reliable stamp of validity for websites providing Orthopaedic Sport Medicine information [19]. We would recommend modification of this code for use in smartphone applications, allowing medical personnel and patients to identify apps which provide quality health information.

The study has some limitations. Firstly, the data was generated from the overview pages and links to publishers’ websites, as funding limitations prohibited us from downloading the full version of the apps. This may have limited our descriptions of these apps and may have potentially underestimated the medical involvement of these apps. However, the reliability of these apps is also dependent on the latest updates noted and the user ratings, which are easily obtainable from overview pages. In addition, this mirrors the situation that potential apps purchasers encounter prior to purchase, and as such we would urge app publishers to provide customers with comprehensive descriptions of author credentials, content source, conflicts of interest on such overview pages. Finally, we appreciate that relevant app identification can be limited by searching for the commonest terms alone. However, this method has been previous utilised in the performance of such studies in other specialities allowing comparison with those apps in orthopaedic sports medicine [1, 5–9, 11–17].

## Conclusion

Orthopaedic Sports Medicine related smartphone apps show great potential to be of significant benefit, providing diverse functionality at your fingertips. However, as medical involvement in their design is low and there is limited use of evidence-based reference material, there are concerns over the quality and content of some apps and recommendation of such resources remains guarded. We advocate the implementation of a validation stamp, increased regulation by regulatory bodies such as the FDA and more importantly, prudence in the purchase of such apps.
