# Erratum to: Smartphone apps for orthopaedic sports medicine – a smart move?

**DOI:** 10.1186/s13102-015-0022-9

**Published:** 2015-11-20

**Authors:** Seng Juong Wong, Greg A. Robertson, Katie L. Connor, Richard R. Brady, Alexander M. Wood

**Affiliations:** 1 University of Edinburgh, College of Medicine and Veterinary Medicine, 7/3 West Nicolson Street, Edinburgh, EH8 9DA UK; 2 Department of Trauma and Orthopaedics, Royal Infirmary Edinburgh, Edinburgh, UK; 3 Department of General Surgery, Kirkaldy, Fife UK; 4 Department of Colorectal Surgery, Western General Hospital, Edinburgh, UK; 5 Department of Orthopaedics, Wansbeck Hospital, Ashington, UK

## Erratum

Since the publication of this article [1], we have been made aware that the declaration of the competing interests of one of the authors was omitted. The ‘Competing interests’ section should have read as follows:

“Richard Brady is the owner of researchactive.com Ltd., a company that provides websites, and develops app and social media solutions for the health care market. No external company was involved in the drafting, editing or analysis within this paper.”
